# Maternal and fetal incidental findings on antenatal magnetic resonance imaging

**DOI:** 10.1007/s00247-021-05074-z

**Published:** 2021-05-27

**Authors:** Lisa Story, Caroline L. Knight, Alison Ho, Sophie Arulkumaran, Jacqueline Matthews, Holly Lovell, Laura McCabe, Megan Byrne, Alexia Egloff, Audrey E. T. Jacques, Jim Carmichael, Jo Hajnal, Andrew Shennan, Mary Rutherford

**Affiliations:** 1 Department of Women and Children’s Health, King’s College London, 10th Floor North Wing, St. Thomas’ Hospital, London, SE1 7EH UK; 2 Fetal Medicine Unit, Guy’s and St. Thomas’ NHS Foundation Trust, London, UK; 3 Centre for the Developing Brain, King’s College, London, London, UK; 4 Department of Radiology, Guy’s and St. Thomas’ NHS Foundation Trust, London, UK

**Keywords:** Fetus, Incidental findings, Magnetic resonance imaging, Pregnancy

## Abstract

**Background:**

Magnetic resonance imaging (MRI) examinations are increasingly used in antenatal clinical practice. Incidental findings are a recognized association with imaging and although in some circumstances their identification can alter management, they are often associated with increased anxiety, for both patient and clinician, as well as increased health care costs.

**Objective:**

This study aimed to evaluate the incidence of unexpected findings in both the mother and fetus during antenatal MRI examinations.

**Materials and methods:**

A retrospective study was undertaken over a five-year period at St.. Thomas’ Hospital in London. Maternal incidental findings were recorded from all clinical reports of all fetal MRIs performed (for clinical reasons and in healthy volunteers) during this period. Fetal incidental findings were recorded only in cases where women with uncomplicated pregnancies were participating as healthy volunteers.

**Results:**

A total of 2,569 MRIs were included; 17% of women had maternal incidental findings. Of these, 1,099 were women with uncomplicated pregnancies who undertook research MRIs as healthy volunteers; fetal incidental findings were identified in 12.3%.

**Conclusion:**

Incidental findings are a common occurrence in antenatal MRI. Consideration should be given to counseling women appropriately before imaging and ensuring that robust local protocols are in place for follow-up and further management of such cases.

## Introduction

Magnetic resonance imaging (MRI) is an increasingly utilized modality in obstetric practice for the further characterization of both fetal and placental abnormalities, as well as for abdominal assessment in women with suspected pathology. It is also used in some circumstances where pathology is suspected but ultrasound (US) images may be suboptimal, such as in cases of raised body mass index (BMI) or oligohydramnios [1]. With the increased diagnostic capability of MRI as a result of improved signal-to-noise ratio and enhanced image quality [2] associated with 3-tesla (T) imaging systems and the use of motion correction post processing techniques [3], unexpected abnormalities, both in the mother and the fetus, are commonly identified. Additionally, with large research studies using MRI, such as those in our own institution including iFIND (Intelligent Fetal Imaging and Diagnosis) [4], the Placenta Imaging Project [5] and the Developing Human Connectome Project [6], unexpected abnormalities may also be detected in otherwise low-risk pregnancies.

Previous studies in adults undergoing imaging have shown that unexpected findings are common, occurring in more than a third of adults undergoing cardiac MRI [7]; although most are unlikely to be of clinical significance, they often need further evaluation to reassure both the clinician and the patient. This may result in anxiety for women and increased health care costs. However, in some circumstances, identifying an unexpected anomaly may also alter the course of a patient’s treatment.

There are a number of studies in the literature regarding incidental findings in adult and paediatric patients, and guidelines have been published with regard to the management of such cases [8–13], but data regarding antenatal MRI, where there is the possibility of unexpected findings in both the mother and fetus, are more limited [14].

This study aims to characterize the incidence of both maternal and fetal incidental findings in a large sample of pregnancies to facilitate enhanced counseling of women before MR imaging as well as to help inform the creation of care pathways for follow-up when abnormalities are identified.

## Materials and methods

A retrospective study was undertaken of all women who had an MRI during pregnancy at the Centre for the Developing Brain at St.. Thomas’ Hospital in London, a tertiary referral service and research centre, between January 2015 and December 2019. Incidental findings were identified from the clinical reports (generated routinely for both clinically indicated and research fetal imaging within the department).

Participants included all pregnant women who underwent a scan for clinical indications who also signed written consent forms that their data could be used for research purposes, those with known fetal/maternal conditions who were undergoing imaging as part of research studies and pregnant women who were participating as healthy controls in research. All women undergoing imaging received information sheets in advance of their MR examination highlighting the possibility of incidental findings being identified. This was then discussed again with the women at the time written consent was obtained just before the scans. All women undergoing imaging were also informed that the department has a standard operating procedure for the follow-up of all incidental findings.

All MRIs were performed on either a 1.5-T (Ingenia; Philips, Best, the Netherlands; or Area XMR; Siemens Healthineers, Erlangen, Germany) or 3-T (Philips Achieva) imaging system. Although the exact imaging protocol varied depending on the research study, for all examinations, an initial T2-weighted image was performed to ensure full coverage of the entire uterus, placenta and cervix. This may or may not include all maternal pelvis structures. However, the views obtained of maternal organs could be in unorthodox planes. All studies should include the fetal head and brain. Most also include the fetal body. Some late gestational age brain studies may not have dedicated fetal body imaging, but the fetal body would have been covered by the initial whole uterus scan. When repeat imaging was undertaken during the same pregnancy, all of the scans were included, but each abnormality was counted once only.

Maternal incidental findings were defined as previously unidentified abnormalities of the maternal structures (including the maternal cervix) and fetal abnormalities were defined as previously unidentified abnormalities of the fetus, amniotic fluid volume, placenta or umbilical cord. In accordance with a previous study by Abdullah et al. [14], incidental findings were categorized as level I, II or III. Level I findings had little to no clinical significance and were deemed as not requiring any further treatment or evaluation [11]. These have to be discussed with the mother and therefore may still cause anxiety. Level II findings had unknown clinical significance and were thought to be clinically relevant during pregnancy and level III findings were of high clinical significance and required urgent follow-up/care planning [14]. Categorisation of these significance levels was decided by a consensus of the multidisciplinary team of authors of this manuscript including radiologists, obstetricians and gynaecologists, and paediatricians.

A specialist perinatal radiologist reviewed all images and the majority were double reported; when a registrar or fellow reviewed the images, these were reviewed and signed off by one of three senior perinatal radiology consultants. Scans with incidental findings were all double reported.

All MRIs during 2015–2019 were included to assess maternal incidental findings. To assess fetal incidental findings, only the MRIs from healthy women, with US fetal anomaly scans reported as normal, who had volunteered for research studies during this period were assessed. In pregnancies with a fetus with a known structural abnormality, whether scanned for clinical or research indications, it was believed that an additional fetal abnormality could be part of an underlying syndrome rather than a true unexpected incidental finding.

Follow-up data were collected when available in order to assess the impact of any incidental findings on care pathways.

## Results

During the study period, 2,569 examinations were undertaken. One hundred and seventy-three women had two scans in the same pregnancy, nine women had three scans and one woman had four. Of the women who had multiple scans in the same pregnancy, 10 women had an additional finding on a subsequent scan (7 cases of a short cervix and 3 cases of mild/moderate hydronephrosis) and 5 had an additional fetal finding (1 case of renal pelvis dilation, 2 matured placenta, 1 fetus was noted to have the cord looped around the neck and 1 had a head circumference >97th centile). When multiple scans were undertaken in the same pregnancy, all scans were included but any incidental findings only counted once. Twenty-eight women were imaged in two separate pregnancies and four women in three separate pregnancies. Of the MRIs undertaken, 1,099 were in healthy volunteers. Imaging details can be seen in Table 1. Scans were reported by 14 different experienced clinicians, including 3 senior radiologists (A.E.T.J., M.R. and J.C.); all other clinicians were perinatal radiology fellows (including authors A.E. and S.A.).

**Table 1:** Imaging details

Maternal age at MRI, mean (SD)	32.8 (5.5)
Fetal gestation at MRI (completed weeks), mean (SD)	28.8 (4.6)
Indication for imaging	**Number (%)**
Research healthy control	1,099 (42.8)
Clinical	1,470 (57.2)
Cardiac defect	318 (12.4)
CNS defect	608(23.7)
Thoracic defect	28 (1.1)
Gastrointestinal defect	35 (1.4)
Unexplained polyhydramnios	3 (0.1)
Congenital infection	28 (1.1)
Urinary tract defect	40 (1.6)
Family history/genetic	45 (1.8)
Fetal growth restriction	25 (1.0)
Placental evaluation	36 (1.4)
Multiple system abnormalities	76 (3.0)
MCDA twins post IUD of one twin	35 (1.4)
MCDA twins TTTS/TAPS	26 (1.0)
Post intrauterine transfusion	2 (0.1)
Musculoskeletal system abnormality	27 (1.0)
Cleft lip/palate	5 (0.2)
Hydrops	4 (0.2)
Neck/facial mass/micrognathia	17 (0.5)
High-risk study groups including high risk of preterm birth, ADHD, depression, trisomy 21, hypertension	111 (4.3)
Incomplete US with suspected abnormality	1 (0.04)
Magnet strength	**Number (%)**
1.5 tesla	1,388 (54)
3 tesla	1,181 (46)

*ADHD* attention-deficit/hyperactivity disorder, *CNS* central nervous system, *IUD* intrauterine death, *MCDA* monochorionic diamniotic, *SD* standard deviation, *TAPS* twin anemia polycythemia sequence, *TTTS* twin-to-twin transfusion syndrome

There were 135 fetuses with incidental findings and, of these, 11 had 2 abnormalities. There were, therefore, 146 findings in total and the overall incidental finding rate in fetuses from uncomplicated pregnancies was 12.3% (Table 2).

**Table 2:** Fetal incidental findings in healthy volunteers

Incidental finding	Number (%)
Neurological	
Level I	
Asymmetry of ventricles >2 mm	15 (9.4)
Cerebellar vermis upward rotation (tegmento-vermian angle >14^o^) with a normally appearing vermis	2 (1.4)
Prominent cisterna magna (anteroposterior) >10 mm	1 (0.7)
Enlarged cerebrospinal fluid space	5 (3.4)
Prominent perivascular spaces in the lentiform nuclei	1 (0.7)
Level II	
Mild ventriculomegaly (10–12 mm)	10 (6.8)
Moderate ventriculomegaly (12-15 mm)	1 (0.7)
Pseudocysts	14 (8.8)
Small transcerebellar diameter (3rd–5th centile)	2 (1.4)
Prominent venous sinus	1 (0.7)
Head circumference <5th centile	1 (0.7)
Head circumference >97th centile	6 (3.8)
Level III	
Polymicrogyria	1 (0.7)
Germinal matrix haemorrhage	1 (0.7)
Cerebellar haemorrhage	1 (0.7)
Subependymal heterotopia	1 (0.7)
Genitourinary	
Level I	
Prominent bladder	1 (0.7)
Level II	
Hydrocele	2 (1.3)
Renal pelvis prominence	15 (9.4)
Level III	
Thorax	
Level I	
Level II	
Level III	
Congenital pulmonary airway malformation	3 (2.1)
Musculoskeletal	
Level I	
Level II	
Talipes	1 (0.7)
Level III	
Abdomen	
Level I	
Level II	
Small stomach	2 (1.4)
Abdominal cyst	2 (1.4)
Ascites	1 (0.7)
Bowel dilatation	1 (0.7)
Level III	
Umbilical cord/placenta/membranes	
Level I	
Mature placental appearance for gestational age	19 (13.0)
2-vessel umbilical cord	3 (2.1)
2 loops of umbilical cord around the neck	5 (3.2)
Level II	
Low-lying placenta	5 (3.1)
Amniotic band	1 (0.7)
Succenturiate lobe	8 (5.5)
Placental infarction	1 (0.7)
Level III	
Miscellaneous	
Level I	
Large for gestation	1 (0.7)
Level II	
Dacrocystocele/nasolacrimal duct cysts	11 (7.2)
Polyhydramnios	1 (0.7)
Level III	
Total level I findings	53 (36.3)
Total level II findings	86 (58.9)
Total level III findings	7 (4.4)

In the 459 pregnancies with maternal incidental findings, 60 women had 2 additional findings, 1 woman had 3 and 1 woman had 4. There were 524 findings in total. The incidental maternal finding rate in women undergoing a fetal MRI was 17% (Table 3).

**Table 3:** Maternal incidental findings

Incidental finding (*n*=524)	Number (%)
Gynaecological	
Level I	
Fibroids <6 cm	35 (6.7)
Nabothian follicles	37 (7.1)
Simple ovarian cyst <5 cm	16 (3.1)
Bartholin’s cyst	5 (1.0)
Uterine abnormality	4 (0.8)
Polycystic ovaries	2 (0.4)
Fluid in vagina (likely physiological)	1 (0.2)
Suspected funneling but long cervix	14 (2.7)
Suspected niche at site of C-section scar	3 (0.6)
Dilated vaginal veins	1 (0.2)
Tortuous ovarian vein	1 (0.2)
Small blood clot in the cervix	1 (0.2)
Level II	
Short cervix (<25 mm)	89 (17.0)
Simple ovarian cyst >5 cm	2 (0.4)
Abnormal signal suggestive of adenomyosis	3 (0.6)
Complex ovarian cyst	7 (1.3)
Fibroids >6 cm or in uterine lower segment	10 (1.9)
Level III	
Open cervix	5 (1.0)
Cord prolapse	1 (0.2)
Urinary tract	
Level I	
Mild/moderate hydronephrosis 5–15 mm	112 (21.4)
Duplex collecting system	14 (2.7)
Urethral diverticulum	1 (0.2)
Unusual configuration of the bladder	1 (0.2)
Malpositioned kidney	1 (0.2)
Bladder trabeculations	1 (0.2)
Level II	
Severe hydronephrosis >15 mm	48 (9.2)
Renal cysts	27 (5.2)
Level III	
Abdomen	
Level I	
Abdominal adhesions	1 (0.2)
Mesenteric cyst	2 (0.4)
Small amount of free fluid noted in abdomen	2 (0.4)
Level II	
Gallstones	2 (0.4)
Liver cyst	6 (1.1)
Splenic cyst	3 (0.6)
Hernia	1 (0.2)
Level III	
Grossly dilated rectum	1 (0.2)
Cutaneous/musculoskeletal	
Level I	
Subcutaneous cyst	3 (0.6)
Lipoma	1 (0.2)
Bone islands on femoral heads	4 (0.8)
Cystic lesion femoral head	5 (1.0)
Oedematous sacroiliac joint	1 (0.2)
Synovitis	1 (0.2)
Perineural cysts	46 (8.8)
Level II	
Degenerative changes in lumbar spine	3 (0.6)
Level III	
Total level I findings	316 (60.3)
Total level II findings	201 (38.4)
Total level III findings	7 (1.3)

Of the 89 women who had a short cervix, less than 25 mm, the median gestational age at MRI was 31 completed weeks (range: 20–38 weeks). Four of these examinations were performed after 37 weeks’ gestation. The gestation at delivery was available for 69 cases, median gestation at delivery was 38^+0^ weeks (range: 26–42 weeks). Forty-eight were delivered at term (70%). Of the 21 delivered at less than 37 weeks’ gestation, the median gestation at delivery was 35^+3^ weeks (range: 26^+0^–36^+6^ weeks). Four of the 21 women were healthy volunteer controls in research studies and 1 woman had a history of depression and was participating in the BIBS (Brain Imaging in Babies) study but otherwise had normal antenatal ultrasound scans. Five women had multiple pregnancies, three of whom were iatrogenically delivered between 36 and 37 weeks’ gestation. One patient had premature rupture of the membranes and was known to be at high risk of preterm delivery before the scan (although the cervical length was unknown before imaging). One woman delivered iatrogenically preterm due to preeclampsia and another due to growth restriction. A further case had a cerclage inserted as a consequence of a short cervix identified on MRI at 21^+5^ weeks’ gestation, but subsequently elected to terminate the pregnancy due to a suspected diagnosis of tuberous sclerosis on a repeat MRI at 30^+0^ weeks’ gestation. The exact details surrounding delivery, other than gestation, were not available for seven women, although all were imaged for fetal abnormalities and it is possible some may have elected to terminate their pregnancies.

**Fig. 1:**
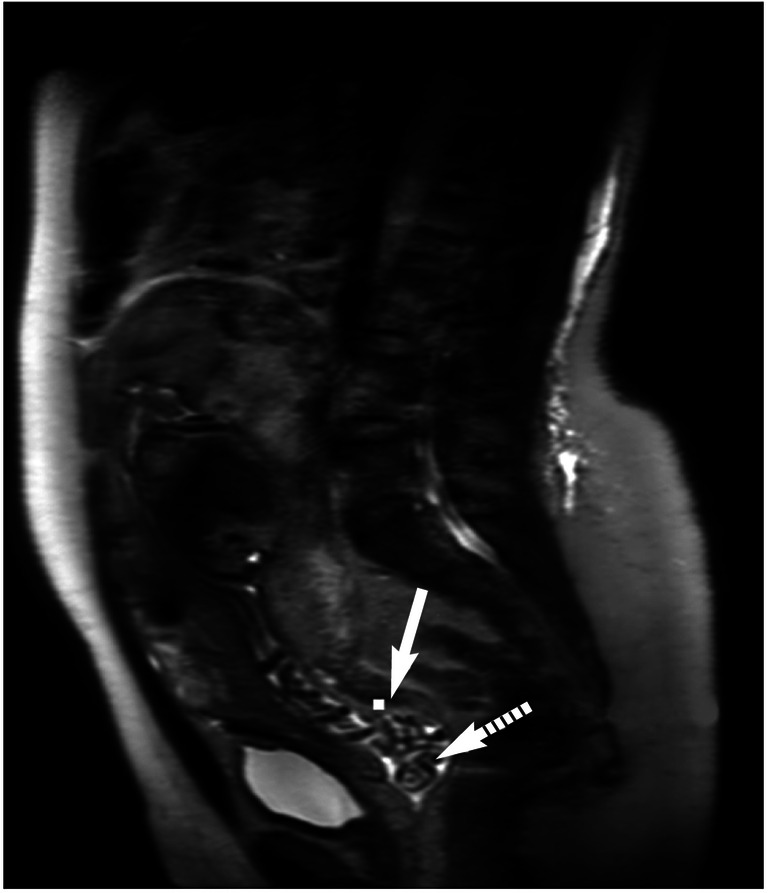
Cord prolapse in a 37-year-old woman imaged at 23^+5^ weeks’ gestation. Sagittal T2 turbo spin echo MR image acquired as part of a research study investigating the development of fetuses at high risk of preterm birth shows the umbilical cord prolapsing into the vagina (*dotted arrow*). The cervix is marked with a *solid arrow*

Five women had an open cervix, one with a cord prolapse noted at MRI (Fig. 1). This woman was participating in a study assessing women at high risk of preterm delivery. She was known to have premature rupture of the membranes, underwent imaging at 23^+5^ weeks’ gestation and delivered at 24^+3^ weeks’ gestation for maternal chorioamnionitis. A neonatal death occurred shortly after delivery. Of the other four women with an open cervix (gestational age range: 19–30 weeks), one was participating in a study of those at high risk for preterm birth and was known to have ruptured membranes, two were twin pregnancies undergoing clinically indicated MRIs (one for cardiac rhabdomyomas and one for ventriculomegaly in one of the fetuses) and the fourth woman was undergoing a fetal cardiac MRI for left atrial isomerism. The median gestational age at delivery was 24^+3^ weeks (range: 19^+6^–36^+3^ weeks).

**Fig. 2:**
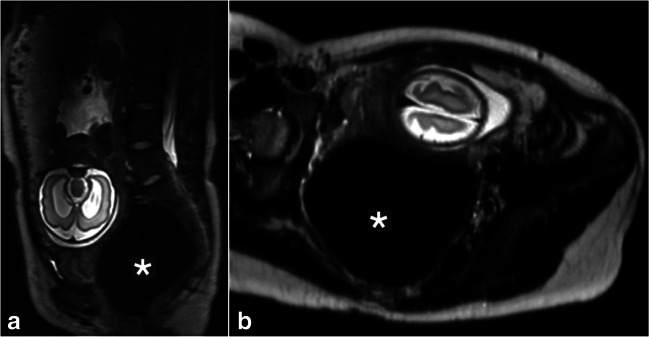
Grossly dilated rectum in a 21-year-old woman imaged at 29^+0^ weeks’ gestation due to bilateral fetal ventriculomegaly found on ultrasound. **a**, **b** Sagittal (**a**) and coronal (**b**) T2 turbo spin echo MR images show rhomboencephalosynapsis with severe ventriculomegaly secondary to aqueductal stenosis and a reduction in cortical folding. The images also show a level III maternal incidental finding of a grossly dilated rectum (*asterisk*)

In addition to women with an open cervix, the other level III maternal finding was a grossly dilated rectum identified on an MRI undertaken at 29^+0^ weeks’ gestation for suspected fetal brain anomalies (Fig. 2). This woman complained of nausea, vomiting and abdominal pain and hence emergency review by the colorectal surgeons was organized; however, the symptoms improved and this was not undertaken. The patient’s care was transferred to another trust for delivery due to the fetal diagnosis of rhomboencephalosynapsis with severe ventriculomegaly secondary to aqueductal stenosis, with a reduction in cortical folding, where paediatric neurosurgical input was required.

**Fig. 3:**
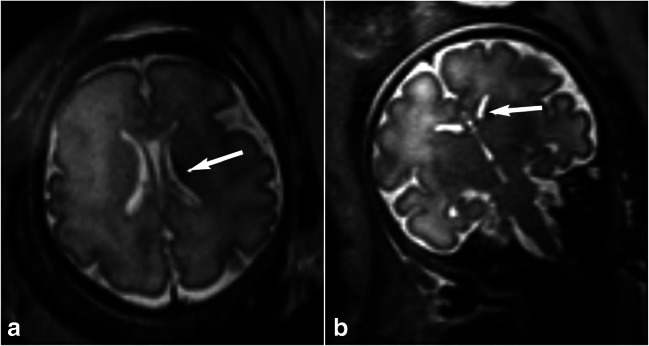
Grade 1 germinal matrix haemorrhage in a 33^+1^ weeks gestational age fetus. **a**, **b** Axial (**a**) and coronal (**b**) dynamic T2 MR images acquired in a healthy volunteer for a research study show a small unilateral focus of T2 hypointensity in the caudothalamic groove suspicious for a grade 1 germinal matrix haemorrhage (*arrows*)

Of the level III fetal findings, there was one case of suspected polymicrogyria. However, the images were subsequently reconstructed using a different algorithm and the gyration was believed to be appropriate for gestational age. The fetus with grade 1 germinal matrix haemorrhage had an uncomplicated neonatal course (Fig. 3). No follow-up data were available for the fetus with a cerebellar haemorrhage. One fetus had suspected subependymal heterotopia and ventricular asymmetry identified on MRI at 28^+6^ weeks’ gestation. A repeat MRI was undertaken at 32^+4^ weeks and no abnormalities were identified.

Three suspected cases of congenital pulmonary airway malformation were identified. One case was reviewed in the Fetal Medicine Unit and the lesion was no longer visible on US in the third trimester. Two patients had chest radiography and computed tomography (CT) performed postnatally and no congenital pulmonary airway malformation was identified in either case. This may represent the natural evolution of the condition.

## Discussion

In this study of a large population of pregnant women undergoing fetal MRI, 17% of women had an incidental maternal finding and 12.3% of uncomplicated pregnancies had an incidental fetal finding.

### Maternal incidental findings

The rate of incidental maternal findings is lower than that in a previous study by Abdullah et al. [14], who reported 79% of 332 pregnancies evaluated had maternal incidental findings: 90.5% level I, 9.2% level II and 0.4% level III. Unlike our study where clinical reports were reviewed retrospectively, they specifically reviewed images for additional findings.

In our study, 60% of findings were level I. These should be reported, as correlation may be required with symptoms for certain conditions (e.g., musculoskeletal findings with pain). Even if follow-up isn’t required immediately (e.g., small simple ovarian cysts [15]), clinicians should still inform women of these findings as they may become relevant in the future. Clinicians can inform women that findings are unlikely to be of any clinical significance and may in some cases be variations of normal.

Thirty-eight percent of women had level II findings, including women with a short cervix. However, their mean gestation at MRI was 31 weeks and cervical length assessment in risk prediction for preterm birth after 32 weeks and in low-risk populations has been shown to be of limited value [16, 17]. Indeed, where follow-up data were available, 70% of these women delivered at term. It is therefore imperative that gestation and additional risk factors for preterm delivery be considered in order to stratify risk appropriately. Other level II findings included large simple and complex ovarian cysts, and renal, splenic and liver cysts, which should be assessed for size, complexity and concomitant haemorrhage, as they are not always completely imaged in the field of view of the fetal MRI [15, 18, 19]. Where significant hydronephrosis is present, correlation with symptoms is essential due to associations with urosepsis and preterm delivery [20, 21]. Large fibroids may pose a risk of postpartum haemhorrhage and, if in the lower segment, may also impact the mode of delivery [22]. Other features, such as adenomyosis, may need evaluation post pregnancy [23]. Level II findings can be significant, so correlation with symptoms is essential. As such, they need to be clearly highlighted to clinicians to enable definitive follow-up.

One percent of women had level III findings. These included five women with open cervices, one of whom was found to also have a cord prolapse and one to have significant rectal dilatation. Identification allowed care planning for the women to optimize outcomes. For such findings, communication needs to be urgent in order to mitigate risks.

### Fetal incidental findings

Published studies on fetal incidental findings are lacking. In our study, 36% were level I. Although they may be of limited clinical significance in isolation, they should still be highlighted to the care team and patient as they may become relevant in the clinical context (e.g., a matured placenta in the presence of a small for gestational age baby could be clinically relevant and help determine if postnatal imaging is required).

Fifty-nine percent of fetal incidental findings were level II. Further antenatal assessment may be required to ensure conditions are not progressive or associated with additional features, like mild or moderate ventriculomegaly [24] or a small head circumference or cerebellum, and to assess the need for additional investigations (e.g., infection screen or chromosomal testing). Further assessment may also be indicated antenatally if MRI was unable to obtain optimal views when it was not the focus of the examination (e.g., prominent renal pelvices). Postnatal follow-up may be appropriate for other findings, such as hydrocele or dacrocystocele [25]. As in the case of talipes, this may simply be positional and represent a false positive; however, the baby still requires postnatal follow-up to correlate the clinical status with imaging findings.

Four percent of fetal incidental findings were level III. These included polymicrogyria, germinal matrix haemorrhage, cerebellar haemorrhage, subependymal heterotopia and congenital pulmonary airway malformation. None of the cases of congenital pulmonary airway malformation was confirmed on later imaging. This may have indicated resolution, but appearances could have been a function of motion artifact or partial volume effects and hence could have been false positives. This could also explain the case of the fetus with suspected polymicrogyria since the findings were not confirmed when motion-corrected images were assessed. These cases were still included in the analysis because they were reported as having pathology, hence requiring care planning and management decisions as well as discussions with the women. However, it does highlight the need for detailed standard operating procedures to deal with suspected incidental findings.

### Limitations

Fourteen clinicians reported the scans within this study, three of whom were senior radiologists who signed off on all reports. Background and training may influence detection and classification of findings, as well as the likelihood of including findings in the report that the reporting radiologist deemed irrelevant to the case. Double reporting is recommended. Only findings within the field of view of imaging could be reported, which varies with the reason for a scan. Unless explicitly mentioned on a referral form, it is unclear whether a finding may have been known before the MRI. Were this the case, they would not be true incidental findings and the overall rate described may be falsely high. Full follow-up data were not available for all of the incidental findings. This is relevant to assessing their true impact.

### Care planning

It is imperative that clinical investigations are requested for appropriate reasons, giving clear questions to be addressed and details of preexisting fetal/maternal conditions. In addition, the possibility of both maternal and fetal incidental findings should be made clear to all patients as a part of the consent process.

Clear guidelines should be in place defining what should be reported as incidental findings, agreed to by both obstetricians and radiologists. More objective assessment with two-dimensional measures may improve classification for some anomalies. In our unit, a reporting template is used to standardize reports and compare values to normal ranges [26, 27].

Local protocols, agreed to by obstetricians, neonatologists, radiologists and general practitioners, are essential in the follow-up of incidental findings. These should include who will take responsibility for arranging follow-up and communicating findings with the patient. Additional resources with associated cost implications may be required [28]. In our unit, it is routine practice to ensure the general practitioner’s and obstetrician’s contact details are recorded before the MRI, which expedites the follow-up process should the need arise.

## Conclusion

Incidental findings are an inevitable consequence of fetal MRI and units undertaking examinations should ensure adequate locally agreed upon protocols and pathways are in place to optimize outcomes for both mother and child.
